# A 10-year microbiological study of *Pseudomonas aeruginosa* strains revealed the circulation of populations resistant to both carbapenems and quaternary ammonium compounds

**DOI:** 10.1038/s41598-023-29590-0

**Published:** 2023-02-14

**Authors:** Marine Pottier, François Gravey, Sophie Castagnet, Michel Auzou, Bénédicte Langlois, François Guérin, Jean-Christophe Giard, Albertine Léon, Simon Le Hello

**Affiliations:** 1 Research Department, LABÉO, 14053 Caen, France; 2 UNICAEN, Univ Rouen Normandie, INSERM DYNAMICURE UMR 1311, CHU Caen, department of microbiology, Normandie Univ, 14000 Caen, France; 3 Service de Microbiologie, CHU de Caen, Avenue de la Côte de Nacre, 14033 Caen Cedex, France; 4 Laboratoire de Bactériologie et Hygiène Hospitalière, CHU de Rennes, 2 Rue Henri Le Guilloux, 35033 Rennes Cedex 9, France; 5 Service d’Hygiène Hospitalière, CHU de Caen, Avenue de la Côte de Nacre, 14033 Caen Cedex, France

**Keywords:** Microbiology, Antimicrobials, Environmental microbiology, Microbial genetics

## Abstract

*Pseudomonas aeruginosa* is one of the leading causes of healthcare-associated infections. For this study, the susceptibility profiles to antipseudomonal antibiotics and a quaternary ammonium compound, didecyldimethylammonium chloride (DDAC), widely used as a disinfectant, were established for 180 selected human and environmental hospital strains isolated between 2011 and 2020. Furthermore, a genomic study determined resistome and clonal putative relatedness for 77 of them. During the ten-year study period, it was estimated that 9.5% of patients’ strains were resistant to carbapenems, 11.9% were multidrug-resistant (MDR), and 0.7% were extensively drug-resistant (XDR). Decreased susceptibility (DS) to DDAC was observed for 28.0% of strains, a phenotype significantly associated with MDR/XDR profiles and from hospital environmental samples (*p* < 0.0001). According to genomic analyses, the *P. aeruginosa* population unsusceptible to carbapenems and/or to DDAC was diverse but mainly belonged to top ten high-risk clones described worldwide by del Barrio-Tofiño et al. The carbapenem resistance appeared mainly due to the production of the VIM-2 carbapenemase (39.3%) and DS to DDAC mediated by MexAB-OprM pump efflux overexpression. This study highlights the diversity of MDR/XDR populations of *P. aeruginosa* which are unsusceptible to compounds that are widely used in medicine and hospital disinfection and are probably distributed in hospitals worldwide.

## Introduction

*Pseudomonas aeruginosa* is a Gram-negative bacterium that is remarkably adaptable and metabolically versatile^1^. Ubiquitous in the environment, it can be found in soil, water, and hospital environments such as sinks and drains^2^. For humans, *P. aeruginosa* is an opportunistic pathogen responsible for various infections, such as hospital-acquired pneumonia, bloodstream, and urinary tract infections^3^, and is notably responsible for severe lung infections of patients with cystic fibrosis (CF). Its presence in humans can be a sign of colonization or chronic or evolving infection^4^. *P. aeruginosa* infections occur particularly in critically ill and immunocompromised patients and are largely health care-associated^5^.

In France, *P. aeruginosa* was the 4th leading microorganism responsible for nosocomial infections^6^ and was shown to increase the total cost of a hospitalization stay^7^. On a larger scale, in 2020, *P. aeruginosa* (6.2%) was the 5th most commonly reported bacterial species from invasive isolates (blood or cerebrospinal fluid)^8^, and the most frequently isolated microorganism in intensive care unit (ICU)-acquired pneumonia episodes^9^ in the European Union/European Economic Area.

One of the challenges of *P. aeruginosa* infections is the difficulty of treatment^10^. Due to their intrinsic resistance to many antibiotics, only eight classes of antimicrobials are currently used against *P. aeruginosa* infections: penicillins with β-lactamase inhibitors, carbapenems, monobactams (aztreonam), cephalosporins, aminoglycosides, fluoroquinolones, phosphonic acids (fosfomycin) and polymyxins (colistin and polymyxin B)^11^. Recent drugs such as ceftolozane-tazobactam, cefiderocol, and imipenem-cilastatin/relebactam were developed^12,13^. However, *P. aeruginosa* can also acquire some resistance by gene mutational processes, which modify the expression and function of chromosomally encoded mechanisms, or by gene acquisition^14^. This problem is such that in 2017, the World Health Organization published a list to prioritize and stimulate research and development of effective drugs, and classified carbapenem-resistant *P. aeruginosa* as a critical priority. Carbapenem resistance constitutes an appropriate marker for multidrug-resistant (MDR), extensively drug-resistant (XDR), and pandrug-resistant (PDR) bacteria because it usually involves a wide range of co-resistance to unrelated antibiotic classes^15^.

It is important to note that if not successfully treated in the acute phase, *P. aeruginosa* can establish chronic biofilm infections, which are difficult or even impossible to eradicate^1,16^. Because biofilms decrease antimicrobial susceptibility, it is important to diagnose early-stage *P. aeruginosa* infections, keeping in mind the high level of resistance cells can possess even in planktonic mode (MDR/XDR profiles).

Carbapenem resistance can be due to chromosomal mutations that can lead to decreased permeability by the outer membrane protein OprD2 deficiency^17^, overexpression of efflux pumps, and hyperproduction of the chromosomal cephalosporinase AmpC or by enzymatic antibiotic modifications^18,19^. Among four efflux pumps described, MexAB-OprM, MexCD-OprJ, MexEF-OprN, and MexXY, the hyperexpression of MexAB-OprM has been found in MDR clinical isolates more frequently^19^. Finally, acquired carbapenemases among *P. aeruginosa* strains are increasingly reported worldwide, mainly concerned with Ambler class B metallo-β-lactamase (VIM and IMP) or Ambler's class A (KPC and GES variants) enzymes^20–22^. The presence of strains carrying highly transferable carbapenemases represents a major health threat, and it is responsible for outbreaks in wards hosting immunocompromised patients. In addition, the overuse and misuse of disinfectants can also lead to decreased susceptibility to medically important antimicrobials due to cross-resistance and/or co-resistance mechanisms^23^. For example, in *P. aeruginosa* antibiotic resistances can be led by exposure to sodium hypochlorite, didecyldimethylammonium chloride (DDAC)^24,25^, or chlorhexidine^26^. These can occur by natural selection or by reinforcement of an acquired resistance mechanism that allows for adaptation to the new environment^24^.

For care facilities, disinfectants are considered the first option against pathogen dissemination^27^. In the European Union, there are approximately 250 different chemical compounds used, either alone or in combination, in disinfectant products including quaternary ammonium-based disinfectants^28^. DDAC is a compound used as a biocide in various applications such as agricultural, food, leisure, and medical equipment, both for private and professional use^25,29^. In health products, it is used for its detergent and disinfectant actions on floors, walls, accessories, examination tables, medicated equipment, and noninvasive medical devices^30^.

The present study focused on *P. aeruginosa*, both patient and hospital environment strains, isolated in a French University Hospital over ten years, 2011–2020, to (1) describe the circulating populations, their genetic diversity, their antimicrobial resistance profiles and resistomes, (2) determine their susceptibility to the DDAC detergent disinfectant, and (3) look for potential epidemiological and resistance mechanisms links between DDAC and antimicrobial resistance.

## Results

### Antimicrobial and DDAC resistance phenotypes

**Figure 1:**
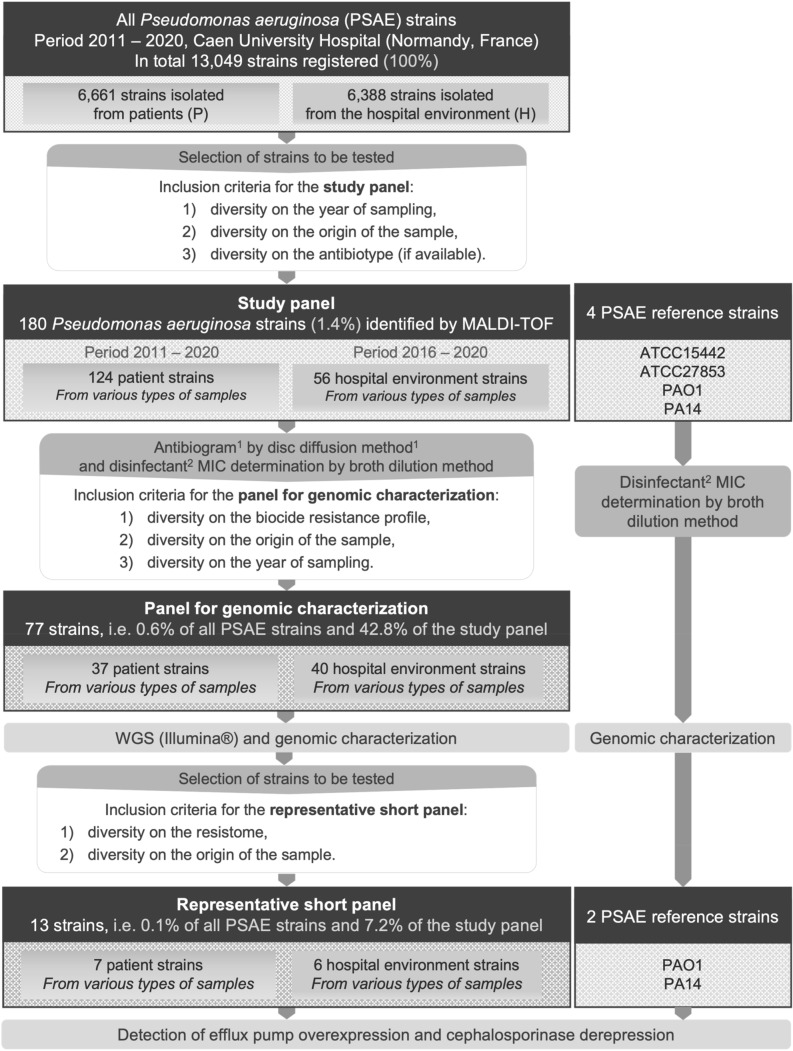
Selection diagram for the study and reference strains. ^1^: 6 classes tested, i.e., 16 antibiotics for hospital use; ^2^: didecyldimethylammonium chloride, ATCC, American Type Culture Collection; H, Strains isolated from the hospital environment; MALDI-TOF, Matrix Assisted Laser Desorption Ionisation/Time of Flight; P, Strains isolated from patients; PSAE, *Pseudomonas aeruginosa*; UHC, University Hospital Center; WGS, Whole Genome Sequencing.

**Figure 2:**
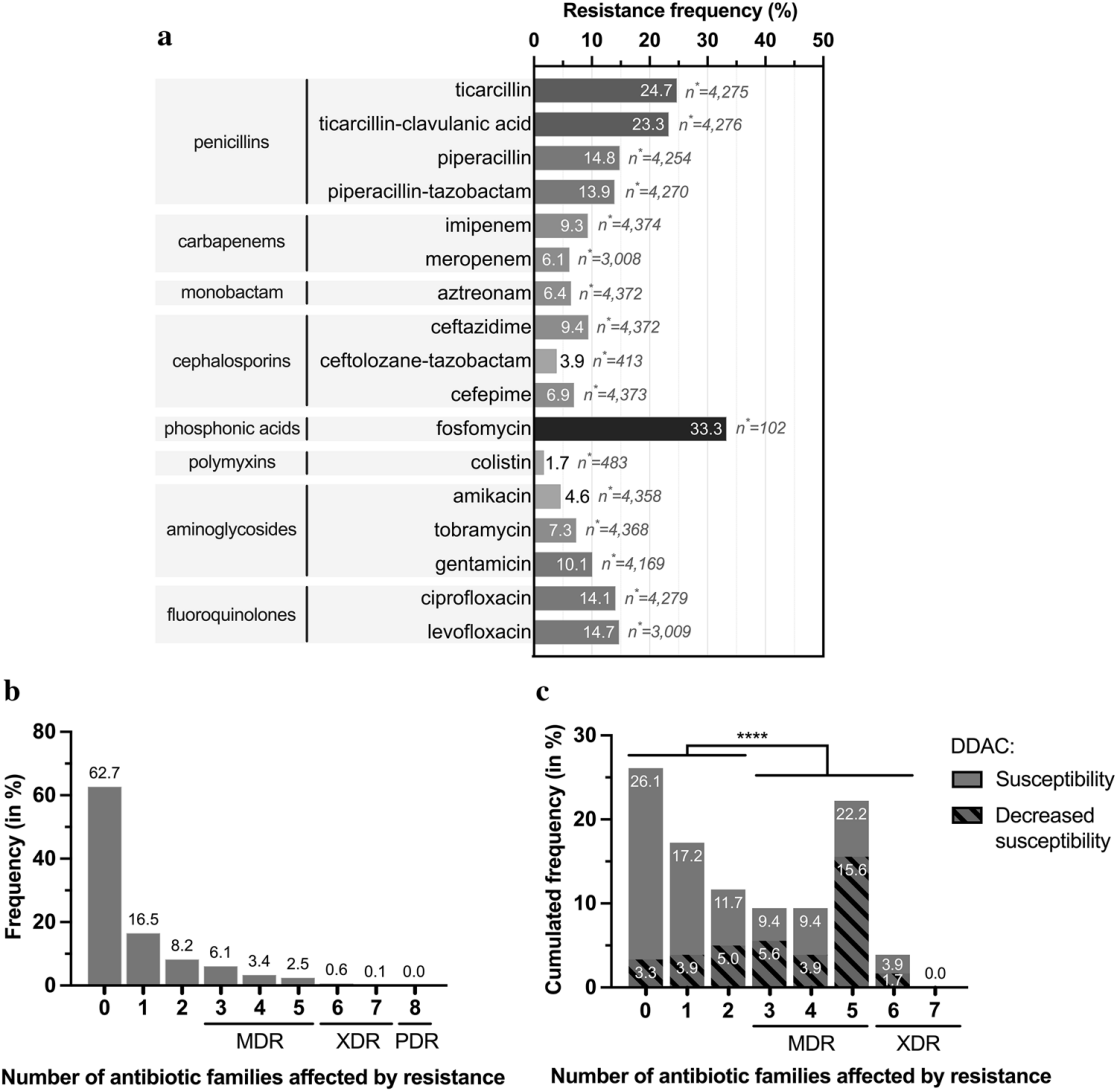
Antimicrobial resistance for hospital strains (n = 4375) and study panel (n = 180). (**a**) Occurrence of antibiotics resistance for all *P. aeruginosa* strains isolated from patients (P strains) tested for antibiotic susceptibility at the University Hospital of Caen (n = 4375). Percentage of strains showing resistance (**b**) to 8 classes of antibiotics for *P. aeruginosa* P strains tested among the total panel (n = 4375), and (**c**) to 7 classes of antibiotics for strains of the study panel (n = 180). (**c**) The hatched areas represent strains that also accumulate DS to the disinfectant DDAC. Fisher’s test for populations independence for DS to DDAC and loss of susceptibility to more than three categories of antibiotics. n*: number of strains tested for the antibiotic; ***: *p* value ≤ 0.0001 after analysis by Fisher’s independence test. DDAC, Didecyldimethylammonium chloride; DS, Decreased susceptibility; MDR, Multidrug-resistant; PDR, Pandrug-resistant; XDR, Extensively drug-resistant.

**Figure 3:**
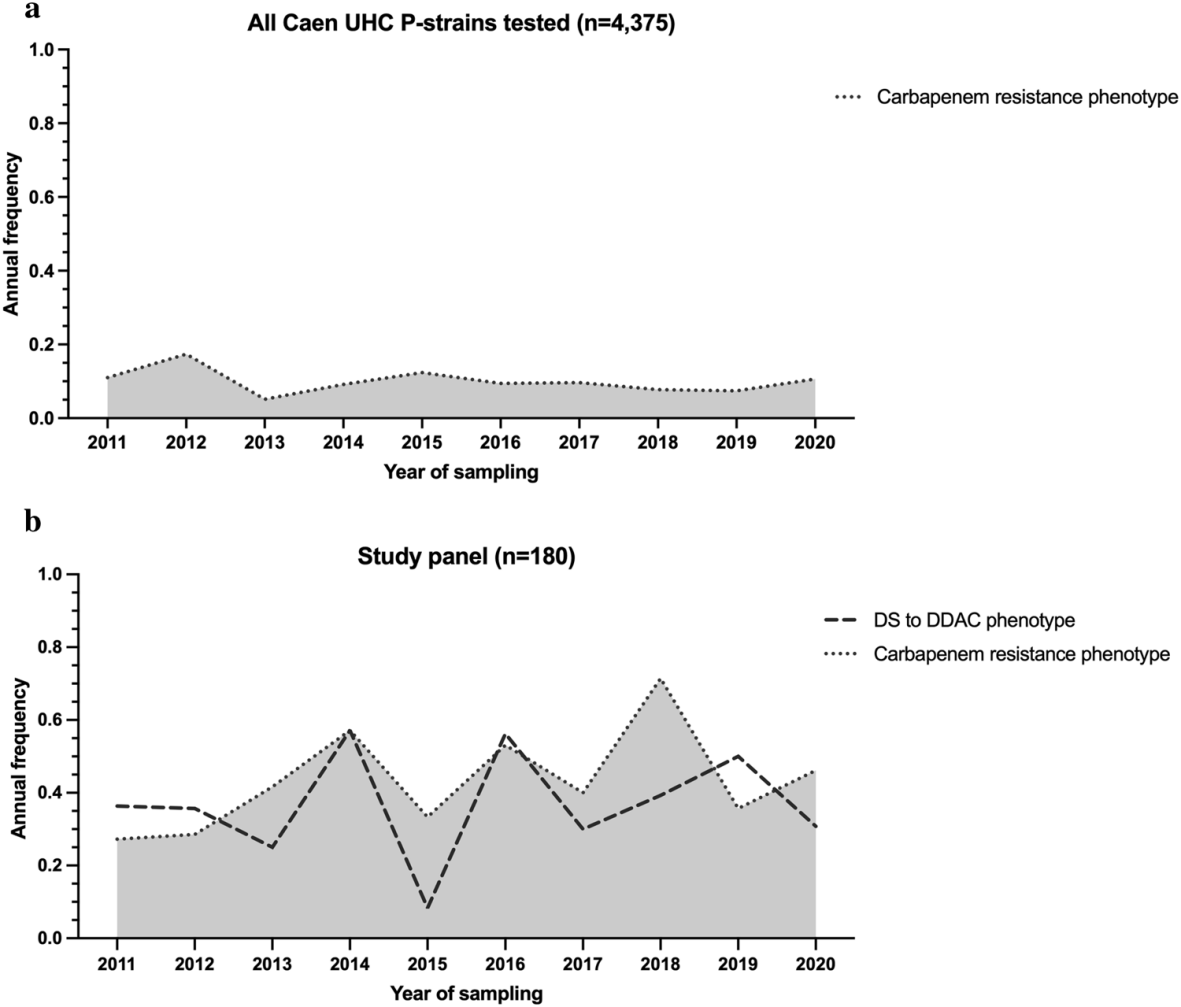
Annual frequency of carbapenem (imipenem and/or meropenem) resistance phenotype for: (**a**) *P. aeruginosa* P strains tested for antibiotic susceptibility at the University Hospital of Caen (n = 4375) and (**b**) the study panel n = 180 (decreased susceptibility to DDAC is indicated).

Between 2011 and 2020, 13,049 strains were isolated at the University Hospital of Caen: 6,661 strains were isolated from patients (P strains, only the first strain from each patient was considered) and 6,388 strains were isolated from the hospital water environment (H strains). Of the P strains, 4,375 were tested for susceptibility to six to eight classes of antibiotics (Fig. 1). Considering MDR strains as having at least one resistance to at least three antibiotic classes, XDR strains as having at least one resistance to all but one or two antibiotic classes, and PDR strains as having no susceptibility to all antibiotic classes tested, 11.9% of strains had an MDR profile, 0.7% had XDR and no strains were classified as PDR (Fig. 2a,b). The proportion of resistance to carbapenems was evaluated at 9.5% (n = 415), with a variable annual frequency from 0.05 [95% CI% 0.03–0.09] in 2013 to 0.17 [95% CI% 0.10–0.28] in 2012 and an average of 0.12 [95% CI% 0.10–0.08] (Fig. 3a). Colistin (1.7%), ceftolozane-tazobactam (3.9%) and amikacin (4.6%) were the most effective antibiotics (> 95%) on clinical *P. aeruginosa* isolated in our university hospital between 2011 and 2020 (Fig. 2b). Surprisingly, no variation in the overall level of resistance was observed between the P strains and H strains.

Of these 13,049 strains, a selection of 180 strains (124 P strains and 56 H strains) constituted the study panel (Fig. 1), all the phenotypes of resistance has been selected with 26.1% (n = 47) of the strains displaying susceptibility to all 16 antibiotics tested (7 classes), 28.9% (n = 52) showing nonsusceptibility to one or two antibiotic classes, 41.1% (n = 74) were considered MDR, and 3.9% (n = 7) as XDR. (Fig. 2c and Supplementary Data 1 [SD] a). Carbapenem resistance was 46.7% (n = 84/180) of the strains, with an average annual expression frequency of 0.43 [95% CI: 0.34–0.53] (Fig. 3b). They were isolated from 13 different sample types, such as sink traps (n = 31/51), urine (n = 17/24), bronchial aspiration (n = 10/16), abscesses (n = 6/11), stools (n = 4/8), sputum (n = 4/11), and blood cultures (n = 4/16). Samples were collected from a variety of wards, mainly the ICU (n = 34/55) and hematology (n = 10/31). Among the carbapenem-resistant *P. aeruginosa* strains, 33 (39.3%) expressed the metallo-β-lactamase VIM-2 enzyme. These last strains were isolated from sink traps (n = 15/51), urine (n = 6/24), bronchial aspiration (n = 5/16), stool (n = 4/8), sputum, blood culture, and catheters (1 for each), and were mainly found in the ICU (n = 21/55) and hematology (n = 4/31) wards. GES-5 was the second most frequent carbapenemase found in strains, with eight cases (SD 2 Part 1).

In addition to assessing the susceptibility of *P. aeruginosa* populations to antibiotics, the susceptibility to a detergent/disinfectant, DDAC, was assessed for the 180 strains in the study panel. MICs ranging from 8 to 128 mg/L were found for this compound, and decreased susceptibility (MIC > 62.9 mg/L) was observed in 38.9% of strains (n = 70/180) (SD 1a). This threshold was chosen because it corresponds to the concentration of DDAC found in a widely used hospital disinfectant. Statistically, the DS to DDAC phenotype was associated with carbapenem resistance (Fisher’s exact test *p* value < 0.0001). More generally, DS to DDAC association increased with the number of drug-resistant strains: for 3.3% (n = 6/180) of strains susceptible to all 7 antibiotic classes tested, for 8.9% (n = 16/180) nonsusceptible to one or two categories of antibiotics, for 25.0% (n = 45/180) and 1.7% (n = 3/180) of strains with MDR and XDR profiles, respectively (Fig. 2c). Even if some strains with DS to DDAC had a low antibiotic resistance level, overall, the DS to DDAC was significantly associated (Fisher’s exact test *p* value < 0.0001) with a high level of antibiotic resistance (nonsusceptibility to more than three antibiotic classes) (Fig. 2c). Furthermore, DS to DDAC was found more often in environmental samples (n = 35, 62.5% of H strains) than in human samples (n = 35, 28.2% of P strains) Fisher’s exact test *p* value < 0.0001 (SD 1b). The annual expression frequency variation is shown in Fig. 3b. To assess the emergence of *P. aeruginosa* strains with DS to DDAC among clinical strains over the last decade, the random inclusion of 10 strains per year confirmed the high frequency, with 28.0% of strains showing DS to DDAC with an MIC range of 64 to 128 mg/L, mainly found in respiratory samples (n = 8/25; 32.0%), surgical pus (n = 4/13; 30.8%), urinary samples (n = 6/21; 28.6%) and digestive samples (n = 4/14; 28.6%).

### Isolation site diversity of the study panel hospital strains and patient information

Among all clinical *P. aeruginosa* strains (n = 6661) isolated over the 2011–2020 period (Fig. 1), the 124 study panel strains were isolated from 21 different wards, mainly from ICU (n = 34, 27.4%), surgery (n = 21, 16.9%), otorhinolaryngology (n = 9, 7.3%), pneumology (n = 9, 7.3%). Most of clinical P strains were isolated from urine (n = 24, 19.4%), bronchial aspiration (n = 16, 12.9%), blood culture (n = 15, 12.1%), sputum (n = 11, 8.9%), and abscess (n = 11, 8.9%). Among all hospital environmental *P. aeruginosa* strains (n = 6388), the 56 H strains were mainly from hematology (n = 23, 41.1%), ICU (n = 21, 37.5%), hepato-gastroenterology (n = 6, 10.7%), and cardiology (n = 5, 8.9%). Sink trap samples represented 91.1% of H strains (SD 3).

Study panel P strains were isolated from patients aged one month to 97 years old, of which 63.7% (n = 79/124) were aged 50 years or older, and the strains were more isolated from men (n = 81/124, 65.3%) than from women. Most of the patients presented altered health status with one or more comorbidities (n = 103/118, 87.3%) and/or immunosuppression (n = 60/116, 51.7%). Regarding their Charlson Comorbidity Index score, more than 61.5% (n = 72/117) had a score ≥ 3, these were patients with an estimated 10-year survival ≤ 77.48%, with multiple comorbidities or one higher-risk comorbidity, and/or elderly patients. Among patients, 12.0% (n = 14) even had a score ≥ 7, which was associated with an estimated 10-year survival ≤ 0.01%. Most of the strains, 81.9% (n = 95/116), were hospital-acquired, and 61.8% (n = 76/123) caused infections during the stay. In total 24 patients died, and the cause of death was associated with *P. aeruginosa* for 8.0% (n = 9/112) of all patients (excluding non-found cases), most of which were hospital-acquired. In total, 60.8% (n = 62/102) of the patients received antipseudomonal antibiotic treatment after the species identification of the strain (Table 1). Untreated patients were associated with various samples including urine (n = 6), sputum (n = 3) and catheters (n = 3). The most prescribed treatments were piperacillin/tazobactam (n = 18, 29.0% of antipseudomonal-treated patients), followed by amikacin (n = 16, 25.8%), ceftazidime (n = 15, 24.2%), ciprofloxacin (n = 15, 24.2%) and imipenem (n = 11, 17.7%). Meropenem was only prescribed for 4.8% (n = 3) of cases (SD 4).

**Table 1:** Demographic and clinical characteristics of patients associated with the P-strains (n = 124) from the study panel.

Demographic/clinical variable	Number of patients	%
Age		
< 20	11/124	8.9
20–29	10/124	8.1
30–39	13/124	10.5
40–49	11/124	8.9
50–59	17/124	13.7
60–69	22/124	17.7
70–79	22/124	17.7
80–89	14/124	11.3
≥ 90	4/124	3.2
Gender		
M	81/124	65.3
F	43/124	34.7
Comorbidities and immune status*		
Comorbidities	103/118	87.3
Immunocompromised	60/116	51.7
Charlson comorbidity index score*		
0	18/117	15.4
1	11/117	9.4
2	16/117	13.7
3	12/117	10.3
4	20/117	17.1
5	15/117	12.8
6	11/117	9.4
≥ 7	14/117	12.0
Acquisition type and infection/colonization status*		
Hospital acquisition	95/116	81.9
Community acquisition	21/116	18.1
Infection	76/123	61.8
Colonization	47/123	38.2
Deceased*		
Death associated with *P. aeruginosa*	9/112	8.0
Antibiotic treatment with an antipseudomonal*		
Treated before strain identification	25/100	25.0
Treated after strain identification	62/102	60.8

*Not found cases were excluded from the total.

### Genomic diversity and resistome analysis

The 77 sequenced strains of the panel (Fig. 1) were distributed into ten serotypes (Table 2). Three major serotypes were found for the P strains (O6 at 35.1%, O11 at 21.6%, and O12 at 13.5%) and H strains (O11 at 42.5%, O6 at 22.5%, and O12 at 10.0%). Other serotypes each represented less than 10% of the population.

**Table 2:** Serotyping and multilocus sequence typing (MLST) of 77 sequenced strains constituting the panel for genomic characterization.

Serotype	Sequence type	High-risk clones	P strains		H strains		Total	
			Number of strains	%	Number of strains	%	Number of strains	%
O11	298	X	2	5	6	15	8	10
	235	X	5	14	2	5	7	9
	309			0	4	10	4	5
	308	X	1	3	3	8	4	5
	316			0	2	5	2	3
Total			8	22	17	43	25	32
O6	233	X	10	27	6	15	16	21
	395		3	8	3	8	6	8
Total			13	35	9	23	22	29
O12	111	X	5	14	4	10	9	12
Total			5	14	4	10	9	12
O4	175	X	3	8		0	3	4
	111	X		0	1	3	1	1
	2407			0	1	3	1	1
	389			0	1	3	1	1
Total			3	8	3	8	6	8
O10	253		2	5	3	8	5	6
Total			2	5	3	8	5	6
O1	27		1	3	1	3	2	3
	207			0	1	3	1	1
Total			1	3	2	5	3	4
O5	244	X	2	5	1	3	3	4
Total			2	5	1	3	3	4
O3	1229		1	3		0	1	1
	274		1	3		0	1	1
Total			2	5		0	2	3
O2	244	X	1	3		0	1	1
Total			1	3		0	1	1
O7	671			0	1	3	1	1
Total				0	1	3	1	1
Overall total			37	100	40	100	77	100

X, Sequence types considered as the global top 10 *P. aeruginosa* high-risk clones based on prevalence, global spread, and association with MDR/XDR profiles and regarding extended-spectrum β-lactamases and carbapenemases^31^; H, Strains isolated from the hospital environment; MLST, Multi locus sequence typing; MDR, Multidrug-resistant; XDR, Extensively drug resistant; P, Strains isolated from patients; VIM-2, Strains producing the class B carbapenemase VIM-2.

**Figure 4:**
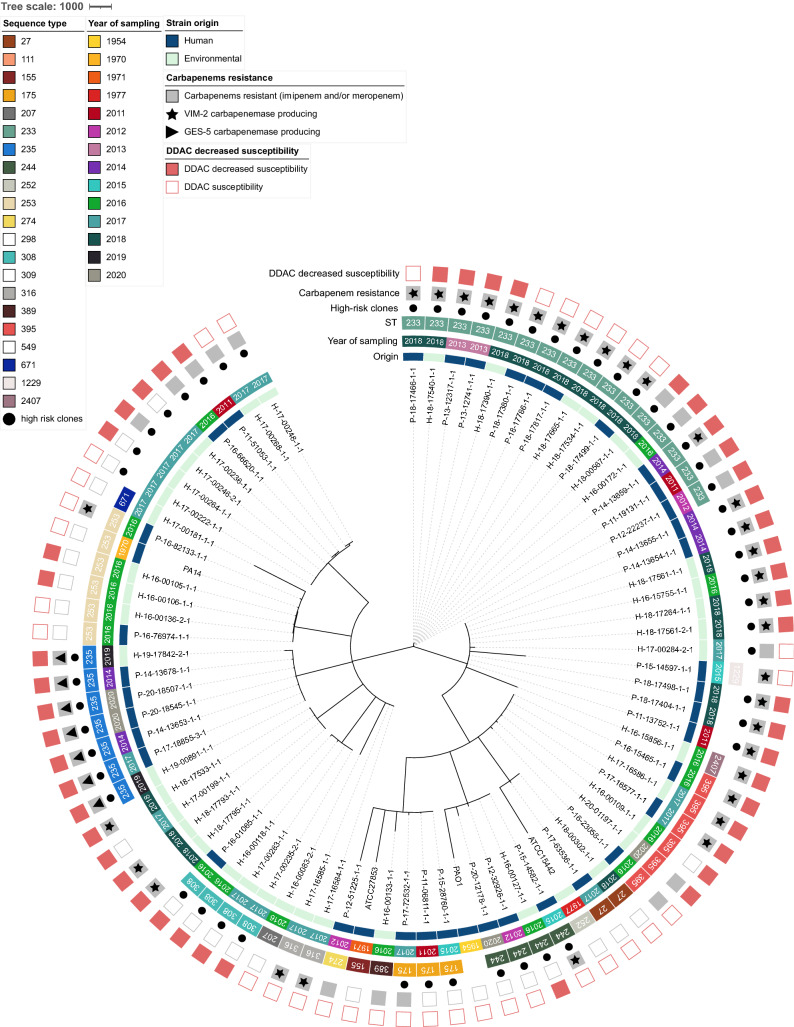
Minimum spanning tree of 77 strains from panel for genomic characterization compared with reference strains (PAO1, PA14, ATCC15442 and ATCC27853). Core genome MLST clustering according to the cgMLST *Pseudomonas aeruginosa* scheme published by Tönnies et al.^32^. The tree was surrounded by the following information: the origin of the strains (patient, P or hospital environment, H), year of sampling, sequence type (ST), carbapenem resistance phenotype, whether it was associated with a VIM-2 or GES-5 carbapenemase, and decreased susceptibility phenotype to DDAC (didecyldimethylammonium chloride). GES-5: strains producing the class A carbapenemase GES-5; VIM-2: strains producing the class B carbapenemase VIM-2.

**Figure 5:**
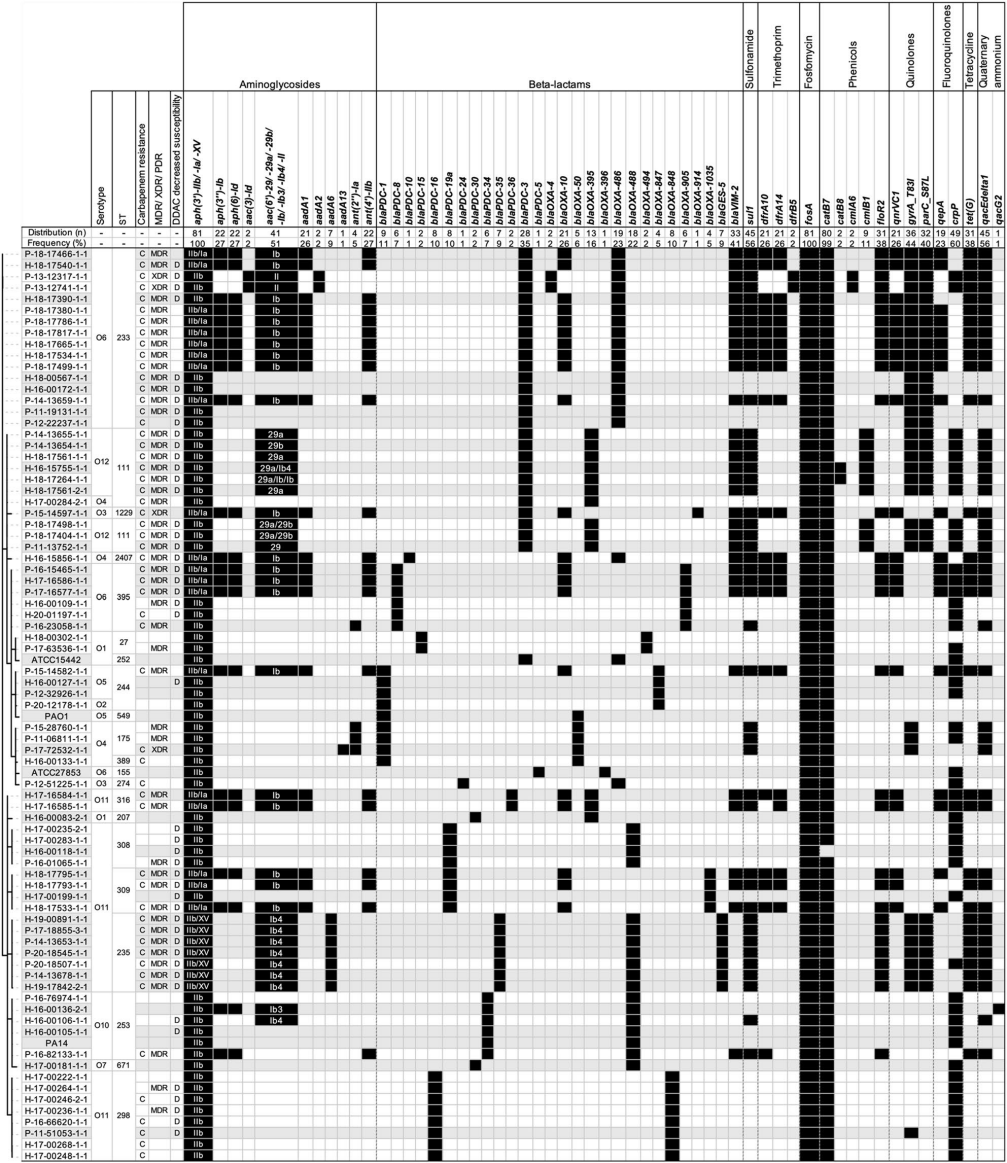
Antimicrobial resistance-associated genes of 77 strains from panel for genomic characterization compared with reference strains (PAO1, PA14, ATCC15442 and ATCC27853). Strains were organized according to the cgMLST minimum distance tree. The serotype (O), the sequence type (ST), the presence of a carbapenem resistance (imipenem and/or meropenem) phenotype, whether the strain was MDR, XDR, or PDR, and the presence of a decreased susceptibility to DDAC phenotype (D) are mentioned. The sub-variants of the *aph(3′)* gene were grouped as well as the sub-variants of *aac(6′)*, in case of co-occurrence of these sub-variants they were indicated and separated by a slash. C, Strains with carbapenems resistance; H, Strains isolated from the hospital environment; P, Strains isolated from patients; MDR, Multidrug-resistant; XDR, Extensively drug-resistant; PDR: pandrug-resistant.

Overall, the panel for genomic characterization was distributed into 18 sequence types (ST). Except for ST111 and ST244, which were associated with O12 and O4 and O2 and O5, respectively, each ST was associated with only one serotype. The most common STs were ST233 (20.8%), associated with serotype O6, ST111 (13.0%), and ST298, associated with O11 (10.4%). The remaining STs were unique or represented between 3.0 and 10.0%. The majority of STs within the P strains were ST233 (27.0%), ST235, and ST111 (13.5% each). The majority of STs of the H strains were ST298 and ST233 (each representing 15.0%) and ST111 (12.5%). This apparent diversity in serotypes and STs was confirmed by the cgMLST results represented in Fig. 4. For cgMLST, of a total of 3,867 loci searched, 3,076 loci were present in the genomes of all strains. Overall, strains diverged in distance from 0 to 2,897 loci and were well distributed according to their origin (human/hospital environment), year of collection, carbapenem resistance, and DDAC resistance (Fig. 5).

Numerous genes conferring antibiotic resistance were found (SD 2 Part 2), some constitutive for *P. aeruginosa* and others acquired. At least one resistance gene was associated with each antibiotic class (Fig. 5). For aminoglycosides, the resistance genes encoded N-acetyltransferases (*aac* variants), aminoglycoside nucleotidyltransferases (*aad* variants), and aminoglycoside O-phosphotransferases (*aph* variants) were found. Regarding resistance to carbapenems, the genes found comprise the carbapenem-hydrolysing class A β-lactamase *bla*_*GES-5*_ and the subclass B1 metallo-β-lactamase *bla*_*VIM-2*_ (Fig. 5). For β -lactamines, the resistance genes comprise the cephalosporin-hydrolysing class C β-lactamase *bla*_*PDC*_ variants and OXA family oxacillin-hydrolysing class D β-lactamases *bla*_*OXA*_. For sulfonamide, only the dihydropteroate synthase *sul1* was found; for trimethoprim its was found dihydrofolate reductases (*dfrA10*, *dfrA14*, and *dfrB5*).

For quinolone resistance, genes coding for the pentapeptide repeat protein QnrVC1, the efflux MFS transporter QepA*,* and modifications of the amino acid sequence in the quinolone resistance determining region (QRDR) due to sequence alterations in position 87 of *parC* and 83 of the *gyrA* genes. Interestingly, the efflux SMR transporters encoding *qacEdelta1* and *qacG2* genes were found but were not statistically associated with the quaternary ammonium resistance (Fig. 5).

### Analysis of the overexpression of efflux pumps and cephalosporinase activity

**Figure 6:**
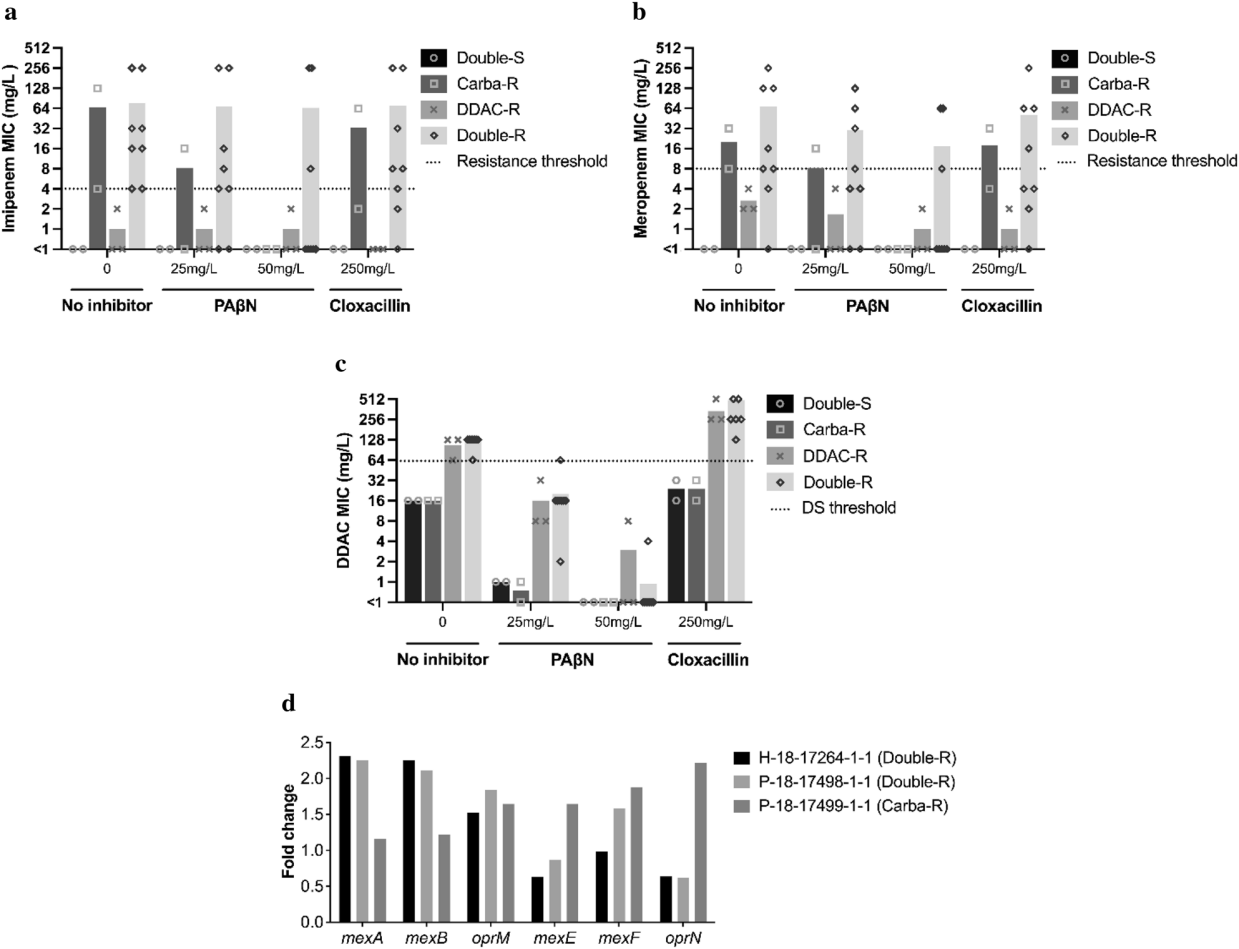
Effect of efflux pumps expression and cephalosporinase activity on susceptibility to carbapenems (imipenem and meropenem) and to DDAC. For the representative short panel strains (n = 13) and 2 reference strains (PAO1 and PA14), MIC variation of the strains to (**a**) imipenem, (**b**) meropenem, and (**c**) DDAC in the absence or presence of PAβN, an efflux pump inhibitor, or cloxacillin, a cephalosporinase inhibitor. A distinction was made between strains with DS to DDAC (DDAC-R strains), carbapenem resistance (Carba-R strains), both phenotypes (Double-R strains), or neither phenotype (Double-S strains). The symbols represent individual MIC values, and the bars represent the average MIC for each strain type. By default, MIC values < 1 were replaced with 0.5. (**d**) Fold-change of expression of *mexA*, *mexB*, *oprM*, *mexE*, *mexF*, and *oprN* genes compared to PAO1 for three strains expressing VIM-2 carbapenemase.

The MICs of imipenem and meropenem were significantly lower in the presence of the efflux pump inhibitor phenyl-arginine-β-naphthylamide (PaβN, especially at 50 mg/L) for all of the 13 hospital-selected *P. aeruginosa* strains, except for those expressing VIM-2 or GES-5 carbapenemase (Fig. 1 and SD 2 Part 3). This was particularly noted for those belonging to the ST111 population, for which no effect was observed. The cephalosporinase inhibitor cloxacillin showed a much more limited inhibitory effect for all the strains tested, resulting in a lower variation of MICs to carbapenems. Regarding susceptibility to DDAC, the effect of the efflux pumps inhibitor PaβN showed at least a twofold reduction in MIC, leading strains categorized as DS to DDAC to a susceptible phenotype. Interestingly, the cephalosporinase inhibitor cloxacillin had the opposite effect of the PaβN, in increasing the level of resistance to DDAC in all strains, including the PA14 and PAO1 reference strains (Fig. 6).

Evaluation of the basal expression of *mexA, mexB, oprM, mexE, mexF,* and *oprN* genes encoding efflux pumps was performed for three selected strains expressing VIM-2. Compared to PAO1 as control, the two strains that cumulated carbapenem nonsusceptibility and DS to DDAC phenotypes overexpressed *mexA, mexB,* and *oprM* genes (fold-change up to 2). In the third strain (non-susceptible to carbapenem but susceptible to DDAC), the *mexE*, *mexF,* and *oprN* genes were overexpressed, which, based on the phenotype of the strain, could not be associated with an increased level of resistance to DDAC (Fig. 6). Taken together, these data strongly suggest that MexA, MexB and/or OprM may play a role in the resistance to DDAC in *P. aeruginosa*, and they are likely to play a role in the carbapenem NS phenotype.

## Discussion

Carbapenem-resistant *P. aeruginosa* strains represent an important factor in the prevalence of hospital-acquired infections worldwide. The mechanism of resistance is carried out by various genetic supports, either through the acquisition of resistance genes on mobile genetic elements (i.e., plasmids) or through mutational processes that alter the expression and/or function of chromosomally encoded mechanisms such as efflux pumps and porins. These strategies can severely limit the therapeutic options for the treatment of serious *P. aeruginosa* infections. This ten-year study conducted in a 1,410-bed teaching hospital in Normandy, France evaluated the proportion of resistance to carbapenems (9.5%), which was mainly supported by the acquisition of carbapenem-hydrolyzing enzymes such as VIM-2 carbapenemase (in 39.3% of carbapenem-resistant strains in the study panel). This prevalence is in accordance with numerous hospital studies pointing out that such of isolates were mainly obtained from patients in ICUs^14^. Furthermore, the prevalence of MDR/XDR strains is an even more serious therapeutic challenge. The occurrence of these strains was increased in nosocomial infections, particularly for patients with altered health and comorbidities, unfavorable Charlson index score, and/or immunocompromised status. Thus, epidemiological outcome studies have shown that infections caused by drug-resistant *P. aeruginosa* are associated with significant increases in morbidity, mortality, duration of hospital stay, associated chronic care, and the overall cost of treating the infection^33^. For treatment efficacy, it is crucial to diagnose *P. aeruginosa* infection/colonization at an early stage in a hospitalized patient. The prescription of the appropriate antibiotic to initiate therapy is essential to optimize the clinical outcome^34^. From our clinical data, we reveal that antipseudomonal prescriptions following species identification were present in only 60% of cases and did not correspond to the most active antibiotics such as colistin, amikacin, ceftolozane-tazobactam, aztreonam or meropenem. The antimicrobial resistance of the *P. aeruginosa* strain populations in this study can be compared with data from a similar study of veterinary strains isolated in Normandy between 1996 and 2020 (from horses, dogs, felins, and bovines)^25^. Among the antibiotics tested, ureidopenicillins, carboxypenicillins, carbapenems, 3rd and later generation cephalosporins, monobactams, levofloxacin and phosphonic acids were considered critically important antibiotic substances not authorized in veterinary medicine, in France since 2016, with exceptions^35^. Legislation that has been harmonized and changed (total ban of these antibiotics in veterinary medicine, modification of the status of levofloxacin and some cephalosporins only) at the European level since 2022^36^. Because of this difference in antibiotics, differences in resistance rates were observed between the hospital and veterinary populations: − 32.7% for phosphonic acids in the veterinary population and − 15.2% for penicillins. However, it was surprising to observe similar rates for other classes of antibiotics: − 5.2% in the veterinary population for monobactams, − 4.8% for cephalosporins and − 1.8% for carbapenems. For other classes of antibiotics, strains isolated from animals showed even more resistance than hospital strains, especially for fluoroquinolones (+ 3.4%) and aminoglycosides (+ 7.2%). It should be noted that these last two classes of antibiotics, along with cephalosporins, are the classes of antibiotics that can be used in veterinary medicine against *P. aeruginosa*. The representation of MDR strains was also close between the hospital and veterinary populations (− 1.8% for animal strains), but no XDR/PDR strains were found in animals.

Recent studies suggest that *P. aeruginosa* strains have nonclonal population structures with few highly successful clusters^22^. This observation was corroborated by our whole genome analyses. Although some genomes had indistinguishable genetic distances, suggesting epidemiologic links, all 77 sequenced strains were well distributed according to their origin (human/environmental), year of collection, or antibiotype. However, among the 18 STs, seven, ST233, ST111, ST298, ST235, ST244, ST308, and ST175, representing 67.5% of the study population. They are considered to be among the top 10 *P. aeruginosa* high-risk clones worldwide based on prevalence, global spread, and association with MDR/XDR profiles and the expressing-carbapenemase strains^31^. Thus, carbapenemase-producing isolates were clearly associated with specific clones, such as ST233, ST111, and ST395, and STs represented once only, all of which express VIM-2 enzymes, and with ST235, which carries the GES-5 enzymes. These carbapenemase-producing strains, which are known to be supported by highly transferable plasmids, were expressed more often in MDR and XDR profiles. Their prevalence increased during the study period, suggesting the role of outbreaks and/or a route of dissemination via environmental reservoirs, also implying that carbapenemase occurrence in *P. aeruginosa* is primarily due to clonal spread. From our other study, similar acquired antimicrobial resistance genes were found in veterinary strains, despite the use of different antibiotics but belonging to similar antibiotic classes. However, no gene associated with the production of a carbapenemase was found to explain the resistance to carbapenems found in the animal strains. Finally, it is interesting to note that in this veterinary study, two strains were associated with ST235^25^, among the top 10 *P. aeruginosa* high-risk clones worldwide.

One point of interest in our study is the MIC determination of *P. aeruginosa* strains to a quaternary ammonium compound, DDAC, that is widely used in hospital disinfectants and as a biocide in various applications. Considering an MIC > 62.9 mg/L, (corresponding to the concentration of DDAC in the commercial disinfectant solution), DS to DDAC existed for 38.9% of strains of the panel and was significantly more associated with MDR and XDR *P. aeruginosa* profiles and more prevalent in the hospital environment (62.5% of them) than in human strains (28.2%). Interestingly, DS to DDAC for P strains trended to be associated in hospital-acquired situation (14.3% in community versus 31.6% in hospital acquisition). Furthermore, the strains showing DS to DDAC circulated throughout the ten-year study period since 2011 and were again estimated at 28.0% by random inclusion among all clinical *P. aeruginosa* strains of our hospital^37^. It was previously described that the DDAC MICs of susceptible strains were between 15 mg/L^38^ and 20 mg/L^39^, which is consistent with our results for a major part of our DDAC-susceptible strains (8–32 mg/L). The phenotype of DS to DDAC was also found in our veterinary study, a phenotype observed in 11.3% of the strains, mainly isolated from horses and associated with respiratory or genital type samples. In this veterinary study, the SD phenotype to DDAC was not associated with an MDR/XDR profile of the strains^25^. Interestingly, in another publication, Goodarzi et al.^40^ showed that out of 92 *P. aeruginosa* hospital strains isolated between 2019 and 2020 (Hamadan City Hospital, Iran), 32.6% had a DDAC MIC ranging from 64 to 128 mg/L as found in the present study. They also tested the efflux pump inhibitor carbonyl cyanide m-chlorophenyl hydrazone (CCCP) at 10 mg/L, and the results showed that 44.6% of their strains had a two-fold to 64-fold reduction in MIC^40^. We also confirmed the important effect of the PaβN inhibitor efflux pump showing a greater decrease in MIC values for DDAC and a lesser decrease for carbapenems (except for carbapenemase-producing strains). We also indicated in our study that this decreased susceptibility may be explained by the intrinsic mechanism of overexpression of the RND efflux pump system such as MexAB-OprM. It is a well-most known drug efflux system constitutively expressed in *P. aeruginosa*, which contributes to intrinsic antibiotic resistance^41,42^ but we need to extent our experiment to all other efflux pump systems. Furthermore, Li et al.^43^ clearly demonstrated that the cephalosporinase inhibitor cloxacillin is a substrate for this pump. This could explain why cloxacillin showed a much more limited inhibitory effect on carbapenem resistance and even increased the DDAC MICs for all the strains tested. Moreover, we can also confidently exclude a potential role of the efflux SMR transporters *qacEdelta1* and *qacG,* even though they were present in many of our studied isolates.

According to Nasr et al., exposure to subinhibitory concentrations of DDAC leads to reduced susceptibility to amikacin, gentamicin, meropenem, and ciprofloxacin^24^. These data reinforce the assumption that antiseptics such as DDAC present in the hospital environment may constitute an important selection pressure leading to the emergence of multidrug resistant strains. Vigilance and monitoring of antimicrobial resistant strains from the close environment of patients appear necessary to prevent hospital-acquired infections. In this context, further studies testing other disinfectants classes as chlorine-based, iodine-based, phenol-based, aldehyde-base disinfectants, alcohol-based, quaternary ammonium-based, and biguanides could be important to lead in the future.

This comprehensive evaluation of the carbapenem resistance mechanisms of *P. aeruginosa* isolates collected over ten years in our hospital highlights the importance of acquired MβL-VIM-2 carbapenemases and chromosomal resistance mediated by the RND efflux pumps system including MexAB-OprM. The assessment of a high proportion of MDR and XDR isolates with DS to a quaternary ammonium compound in our hospital environment is worrisome. This phenomenon was not explained by the dissemination of limited specific strains but appears as the consequence of cumulative mechanisms of resistance regardless of the *P. aeruginosa* population. *P. aeruginosa* strains resistant to compounds that are widely used in medicine and hospital disinfection are probably distributed in hospitals worldwide and can severely limit the therapeutic options for the treatment of serious infections.

## Materials and methods

### *P. aeruginosa* bacterial strains

#### Reference strains

Four well-described and genome-available reference strains were used in the present study, ATCC27853 and ATCC15442, obtained from the American Type Culture Collection (ATCC), and PAO1 and PA14, from the collection of Institut Pasteur (Paris, France). Strain ATCC15442 is recommended for disinfectant susceptibility testing^44^, strain ATCC27853 is the *Pseudomonas* spp. reference for antibiotic susceptibility testing^45^, PAO1 is the reference genome for the *P. aeruginosa* species^46^ and strain PA14 is a highly virulent isolate representing the most *P. aeruginosa* common clonal group worldwide contrary to PAO1^47^.

#### Hospital strains and study panel

Data on all *P. aeruginosa* strains from Caen University Hospital, a 1,410-bed teaching hospital in Normandy, France, were extracted from the laboratory management system (TD-Synergy, Montbonnot-Saint-Martin, France) for human strains (only the first strain isolated from each patient, without distinguishing between strains sought for carriage or diagnosis), and from annual reports of the hospital hygiene ward for hospital environment strain^48^. In total during the period of 1 January 2011 to 31 December 2020, 13,049 *P. aeruginosa* strains were isolated; 6,661 strains (51.0%) came from patients (P strains) and 6,388 (49.0%) from the hospital water environment (H strains) (Fig. 1 and SD 5). All were stored at room temperature in an agar medium, or at − 80 °C on brain–heart infusion medium (BHI; bioMérieux, Marcy-l’Étoile, France) with 15% glycerol (VWR, Radnor, Pennsylvania, USA). Among them, 180 strains were selected retrospectively based on the year and hospital ward isolation and the antibiotype to constitute the “study panel” (Fig. 1 and SD 2 Part 1). Of these, 124 were from patients (without distinguishing between strains sought for carriage or diagnosis) over the 2011 to 2020 period, and 56 were from the hospital environment over the 2016 to 2020 period (before 2016, H strains could not be included because they were not conserved). All were subcultured on tryptic soy broth (TS; Bio-Rad, Hercules, California, USA) and then stored on 15% glycerinated BHI at − 80 °C. The species identification was determined by matrix-assisted laser desorption ionization-time of flight (MALDI-TOF) mass spectrometry (Microflex; Bruker Daltonik, Bremen, Germany). Then, from the study panel, 77 strains were selected based on the resistance profile to represent the variability of antibiotic and disinfectant resistance patterns, the origin of the sample, and the sampling year to constitute the panel for genomic characterization (SD 2 Part 2). Thirty-seven of them were from patients, and the other 40 were from environmental samples. Among them, 13 strains (6 from patients and 7 from the hospital environment, with various resistomes) were selected to determine the overexpression of efflux pumps and cephalosporinase (AmpC), and it constituted the "representative short panel" (SD 2 Part 3).

In parallel to this study, 100 strains of *P. aeruginosa* (10 per year) were randomly selected from the 6661 P strains of Caen UH, to estimate the frequency of nonsusceptibility to DDAC (data not shown).

#### Strains and patient data (SD 2 part 1)

Available data were extracted for all 180 strains from the laboratory software (TD-Synergy TECHNIDATA, Montbonnot-Saint-Martin, France), including the year, origin and type of sampling, hospital ward, and carbapenemase-expressing status. In addition, patient information was retrospectively collected, including the patient gender, age, presence of comorbidities, immune status, whether it was a *P. aeruginosa* infection or colonization, whether or not death occurred (*P. aeruginosa*-related or not), and antipseudomonal antibiotic therapy administered during hospitalization (before and after *P. aeruginosa* identification). According to the European Committee on Antimicrobial Susceptibility Testing (EUCAST), the following drugs were considered active against *P. aeruginosa* for medical use: antipseudomonal penicillins (ticarcillin, ticarcillin-clavulanic acid, piperacillin, and piperacillin-tazobactam), carbapenems (doripenem, imipenem, imipenem-relebactam, meropenem, and meropenem-vaborbactam), monobactams (aztreonam), 3rd- and 4th-generation cephalosporins (cefepime, cefiderocol, ceftazidime, ceftazidime-avibactam, and ceftolozane-tazobactam), aminoglycosides (amikacin, gentamicin, netilmicin, and tobramycin), fluoroquinolones (levofloxacin, ciprofloxacin), fosfomycin and polymyxins (colistin and polymyxin B)^49^. The P strain *P. aeruginosa* acquisition type was also determined: community (i.e., the acquisition occurred before or during the first 48 h of hospitalization) or hospital acquired. The Charlson comorbidity index score was calculated using the available tool at https://www.rdplf.org/calculated^50^.

### Antimicrobial susceptibility testing (AST)

#### Antibiotic susceptibility testing

Among the 6661 P strains, ASTs were available for 4375 *P. aeruginosa* strains against 16 antipseudomonal drugs categorized into 8 classes: penicillin (ticarcillin, ticarcillin-clavulanic acid, piperacillin, piperacillin-tazobactam), carbapenems (imipenem, meropenem), monobactams (aztreonam), cephalosporins (cefepime, ceftazidime, ceftolozane-tazobactam), phosphonic acid (fosfomycin), polymyxins (colistin), aminoglycosides (amikacin, tobramycin, gentamicin) and fluoroquinolones (ciprofloxacin, levofloxacin). Disk diffusion method was performed for all except for colistin and fosfomycin, tested by broth microdilution by VITEK^®^ 2 (bioMérieux, Marcy-l’Étoile, France) or by the Sensititre™ Vizion™ System (Thermo Fisher Scientific, Waltham, Massachusetts, USA). Interpretation of values according to the guidelines of the Comité de l’antibiogramme de la Société Française de Microbiologie (CASFM) each year, from 2011 to 2020. The CASFM guidelines differ from the EUCAST guidelines until 2013 (inoculum, incubation time, and temperature), then the hospital CASFM will follow the EUCAST guidelines. The data were gathered to estimate the percentage of resistance (R) for each antibiotic and classification in multidrug-resistant (MDR), or extensively drug-resistant (XDR) profiles.

For the 180-strain study panel, antibiotic susceptibility testing was performed again for 16 antipseudomonal antibiotics (Bio-Rad, Hercules, California, USA) in association or not with a β-lactamase inhibitor that are distributed in 7 distinct classes: the same as described above, except for polymyxins which were excluded. MIC for colistin was not performed. Antibiotic susceptibility testing was performed on Mueller–Hinton agar (Becton Dickinson, Franklin Lakes, New Jersey, USA) following the EUCAST guidelines for *Pseudomonas* spp. disk diffusion method^45^. Following the 2021 edition of the EUCAST breakpoint tables for interpretation, breakpoints were set^49^. Strains showing resistance (R) in at least three antibiotic classes were considered MDR. Those with remaining susceptibility (“Susceptible, standard dose”, S and “Susceptible, increased exposure”, I) in one or two antibiotic categories were considered XDR. Pan resistance could be defined as resistance to all eight antibiotic classes^51^.

#### Quaternary ammonium susceptibility testing

Susceptibility to the detergent/disinfectant DDAC was evaluated for the 180-strain study panel by determining the minimum inhibitory concentration (MIC) using the reference liquid microdilution method^52^ in cation-adjusted Mueller–Hinton broth with N-Tris(hydroxymethyl)methyl-2-aminoethanesulfonic acid (Thermo Fisher Scientific, Waltham, Massachusetts, USA). The DDAC was tested in the concentration range from 0.5 to 1024 mg/L (successive two-fold dilutions), three times for each strain. By relying on the concentration of DDAC in the disinfectant solution according to the manufacturer’s instructions, the threshold for decreased susceptibility (DS) to DDAC in this study was set at MIC > 62.9 mg/L, equivalent to 0.00629%.

Furthermore, for the 100 randomly selected *P. aeruginosa* strains between 2011 and 2020 the MIC of DDAC was determined under the same technical conditions^37^.

### Whole-genome sequencing and bioinformatic analysis

For the reference strains, ATCC27853 and PA14 genomes were obtained from the European Nucleotide Archive (ENA) database with accession numbers CP015117 and ASWV01000001, respectively, while the ATCC15442 and PAO1 sequences were obtained from GenBank with accession numbers GCF_000504485.1 and GCA_000006765.1, respectively.

The 77 strains were sequenced by the “Plateforme de Microbiologie Mutualisée P2M” (Institut Pasteur, Paris, France). A MagNA Pure 96 instrument (Roche Diagnostics, Meylan, France) was used for DNA extraction, a Nextera XT library kit (Illumina Inc., San Diego, USA) was used for NGS library construction, and a NextSeq500 (Illumina Inc., San Diego, USA) was used for sequencing. FastQC V0.11.0^53^ and MultiQC V1.9^54^ software were used for quality control checks on raw sequence data. The paired-end reads were preprocessed (filtered and trimmed) using fqCleanER (https://gitlab.pasteur.fr/GIPhy/fqCleanER), with a minimal read size of 70 bp and a Phred quality score of 28. De novo assembly was performed using SPAdes 3.12^55^ with a 50X minimum average sequencing depth, and Quast software V5.0^56^ was used for final assembly quality checks.

Species identifications based on the sequences have been validated with Ribosomal Multilocus Sequence Typing (rMLST), available at https://pubmlst.org/^57^. In silico, *P.* *aeruginosa* strain serotyping was performed using PAst1.0 software, available at https://cge.cbs.dtu.dk/services/PAst-1.0/^58,59^. Then, multilocus sequence typing (MLST) was performed on the sequence variation of 7 housekeeping genes using MLST2.0 software (version 2.0.4, 2019/05/08; database version 2021/10/04). This software uses the MLST allele sequence and profile obtained from PubMLST.org^58,60^. Finally, the core genome MLST was determined based on the *P. aeruginosa* MLST scheme targeting 3,867 loci (available on cgmlst.org at https://www.cgmlst.org/ncs/schema/16115339/locus/, database version 2021/05/26)^32^. Chewbacca software version 2.8.5^61^ was used for the cgMLST scheme conversion and allele calling. Finally, a neighbor-joining tree based on cgMLST was visualized with iTOL v6.3.3^62^. The nucleotide sequences were submitted to AMRFinderPlus analysis (version 3.10.30, database version 2022–05-26.1)^63^ with a minimal identity of 80% and minimal coverage of 50% to identify antimicrobial resistance genes and known resistance-associated point mutations. All 77 assembled genomes were deposited in the BioProject PRJNA884650.

### Analysis of the overexpression of efflux pumps and cephalosporinase activity

Imipenem, meropenem, and DDAC MICs values were determined in the presence/absence of the efflux pump inhibitor PaβN (at 25, 50 and 100 mg/L) and the cephalosporinase (AmpC) inhibitor cloxacillin (at 250 mg/L) separately. Susceptibility testing was performed by broth microdilution method^52^, as previously described in the study, for the 13 strains of the representative short panel and 2 reference strains (PAO1 and PA14). In addition, the expression of the *mexA, mexB, oprM, mexE, mexF,* and *oprN* genes was determined by quantitative real-time PCR. Total RNA was extracted from bacterial cells to the late exponential phase using the Direct-Zol RNA miniprep kit (Zymo Research, Irvine, California, USA), and the residual chromosomal DNA was removed using a Turbo DNA-free kit (Life Technologies, Carlsbad, California, USA). A NanoDrop One spectrophotometer (Thermo Fisher Scientific, Waltham, Massachusetts, USA) was used for quantification, and cDNA was synthesized from total RNA using a QuantiTect reverse transcription kit (Qiagen, Hilden, Germany) following the manufacturer’s guidelines. The transcript levels were determined by the ΔΔCT method (CT is the threshold cycle) using the expression of the housekeeping *gyrB* gene as a reference transcript. The level of transcription measured for the PAO1 reference strain was used to determine the expression ratios for the strains of interest. The sequences of the primers associated with each gene are listed in SD 6.

### Statistical analysis

All statistical tests were performed with GraphPad Prism version 9.0.0 for macOS (GraphPad Software, San Diego, California USA). Independence tests of populations using Fisher’s exact test were performed successively to determine if the DS to DDAC was linked to nonsusceptibility of strains (MDR or XDR phenotypes) and/or if the DS to DDAC was linked to carbapenem resistance. A chi-square test was performed to compare the distribution of DS to DDAC according to the strain origin. Carbapenem resistance average annual expression frequency was obtained by averaging annual expression frequency obtained for each year between 2011 and 2020, and 95% confidence intervals were calculated by the Wilson/Brown method.

### Ethics statement

This study has been conducted in compliance with the Helsinki Declaration (ethical principles for medical research involving human subjects) and in accordance with the guidelines of research board of our teaching hospital, Caen, France. Ethic committee of CHU Caen Normandie reviewed and approved the study number ID 3784. It was a non-interventional study: specimens used in this study were part of the routine patient management without any additional sampling. Furthermore informed consent was obtained from all subjects and/or their legal guardian(s).

### Informed consent

Informed consent was obtained from all subjects and/or their legal guardian(s).

## Supplementary Information

Supplementary Information 1.

Supplementary Information 2.

## Data Availability

The datasets generated during and/or analysed during the current study are available in the repository, https://doi.org/10.6084/m9.figshare.c.6293046. All the DNA sequences of the present study can be downloaded in the BioProject PRJNA884650.
