# Supplementary material for: A 10-year microbiological study of Pseudomonas aeruginosa strains revealed the circulation of populations resistant to both carbapenems and quaternary ammonium compounds
Source: Sci Rep. 2023 Feb 14;13:2639. doi: 10.1038/s41598-023-29590-0 (PMC9929048; doi:10.1038/s41598-023-29590-0)
Supplement: Supplementary file 2 — Supplementary Information 2. [file 41598_2023_29590_MOESM2_ESM.docx]

Supplementary Material


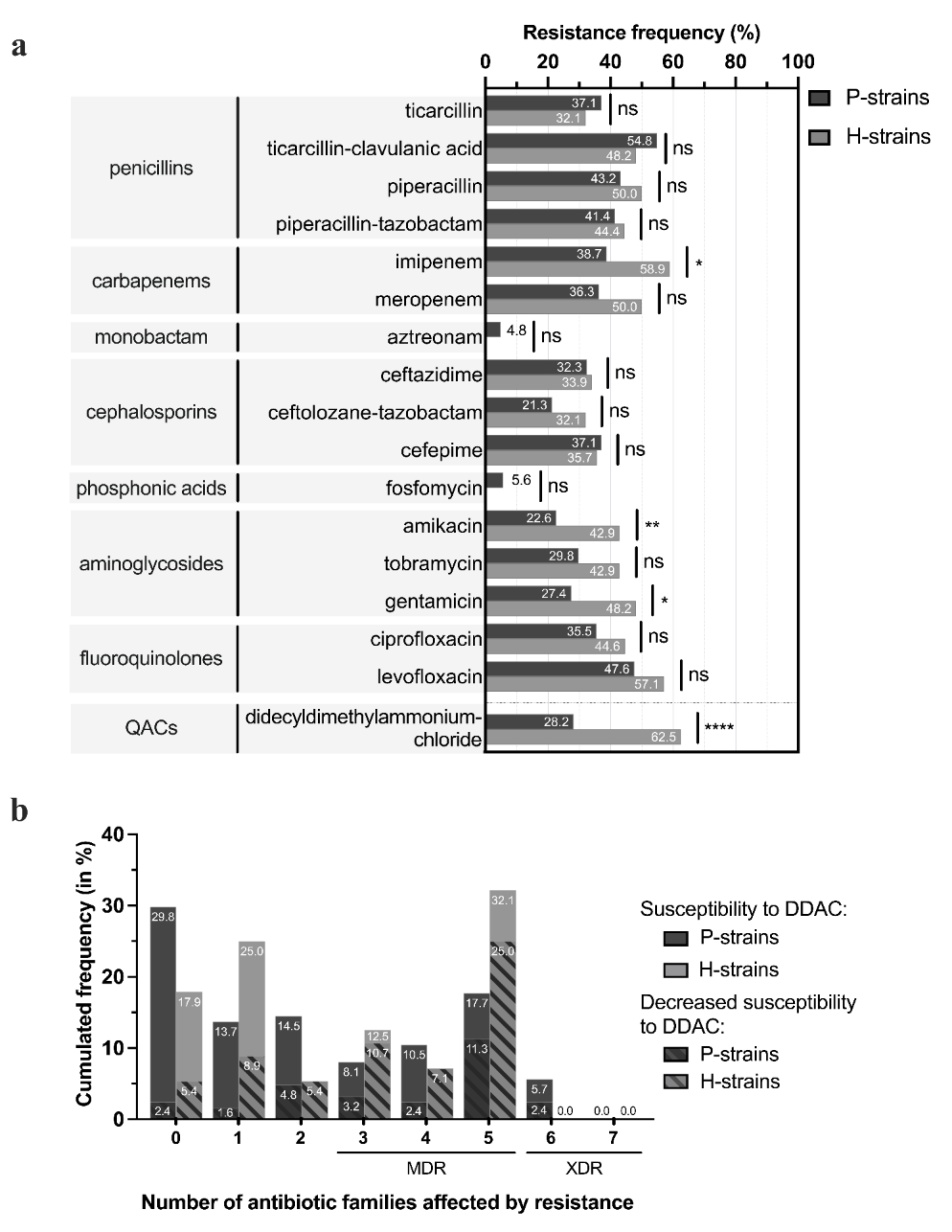
**Supplementary Data 1. Antimicrobial resistance for the study panel (n=180).** (**a**) Occurrence of antibiotics resistance for patient strains (n=124) and hospital environment strains (n=56) of the study panel. For antibiotics, resistance breakpoints were set according to the CASFM/EUCAST 2021, or 2019 edition if a breakpoint was not available, and for DDAC, the DS breakpoint was the concentration in the disinfectant solution following the manufacturer's instructions. Fisher’s test for populations independence for human and environmental hospital strains resistance frequency. (**b**) Percentage of strains of the study panel (n=180) showing resistance to 7 classes of antibiotic. The hatched areas represent strains that also accumulate DS to the disinfectant DDAC. Fisher’s test for populations independence for DS to DDAC and loss of susceptibility to more than three categories of antibiotics. ns: p-value > 0.05, *: p-value ≤ 0.05, **: p-value ≤ 0.01, and ****: p-value ≤ 0.0001 after analysis by Fisher’s independence test. DDAC: didecyldimethylammonium chloride; DS: decreased susceptibility; H: hospital environment; MDR: multidrug-resistant; P: strains isolated from patients; XDR: extensively drug-resistant.

**Supplementary Data 2.** **Characteristic of the strains used in the study panel (Part 1), in the panel for genomic characterization (Part 2) and in the representative short panel (Part 3).**

Please see the joined Excel file named Supplementary_data2.xls


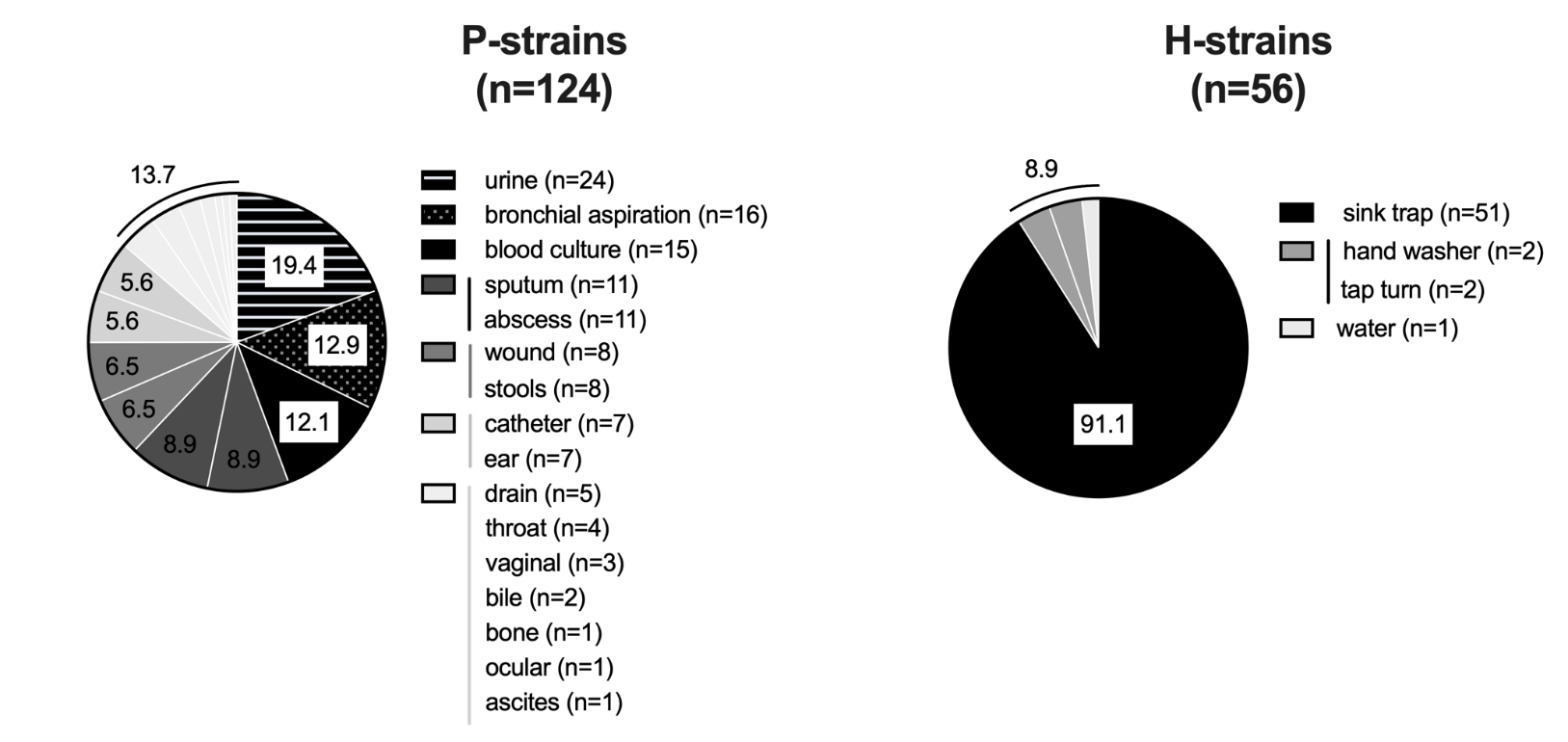
**Supplementary Data 3. Distribution by sample type of the study panel (n=180).** H: strains isolated from the hospital environment; P: strains isolated from patients.

**Supplementary Data 4. Temporal distribution: of all *Pseudomonas aeruginosa* strains isolated at Caen UHC over 2011-2020, the study panel, and of genomic characterization panel.**

|  |  | **All *P. aeruginosa* strains isolated**  **(N=13,049)** | | **Study panel**  **(n=180)** | **Panel for genomic characterization (n=77)** |
| --- | --- | --- | --- | --- | --- |
|  | Sampling year | Strains/year | **Average strains/year** [IC95%] | Strains/year | Strains/year |
| P-strains | **2011** | 630 | **666.1** [615.1-717.1] | 11 | 4 |
|  | **2012** | 526 |  | 14 | 3 |
|  | **2013** | 576 |  | 12 | 2 |
|  | **2014** | 655 |  | 14 | 5 |
|  | **2015** | 659 |  | 12 | 3 |
|  | **2016** | 737 |  | 12 | 6 |
|  | **2017** | 715 |  | 11 | 4 |
|  | **2018** | 725 |  | 16 | 7 |
|  | **2019** | 722 |  | 10 | - |
|  | **2020** | 716 |  | 12 | 3 |
|  | **Total** | 6,661 | - | 124 | 37 |
| H-strains | **2011** | 283 | **638.8** [389.2-888.4] | - | - |
|  | **2012** | 284 |  | - | - |
|  | **2013** | 291 |  | - | - |
|  | **2014** | 310 |  | - | - |
|  | **2015** | 589 |  | - | - |
|  | **2016** | 1,026 |  | 20 | 11 |
|  | **2017** | 542 |  | 19 | 14 |
|  | **2018** | 989 |  | 12 | 12 |
|  | **2019** | 957 |  | 4 | 2 |
|  | **2020** | 1,117 |  | 1 | 1 |
|  | **Total** | 6,388 | - | 56 | 40 |
| **General total** | | 13,049 | **1,304.9** [1,012.1-1,597.7] | 180 | 77 |

**Supplementary Data 5. The sequence of oligonucleotide primers.**

| Target gene | Type | Sequence 5’-3’ | Amplicon size (bp) |
| --- | --- | --- | --- |
| *mexA* | Forward | CTGGAAGGTCGCCTCGAATT | 155 |
|  | Reverse | AGGATGGCCTTCTGCTTGAC |  |
| *mexB* | Forward | CGATCGTGATGACCTCCCTG | 146 |
|  | Reverse | TACCCAGAAGATCGCCAGGA |  |
| *oprM* | Forward | CCTGGACTACGCGAAGATCC | 169 |
|  | Reverse | GAGCTGGTAGTACTCGTCGC |  |
| *mexE* | Forward | GGTCTACGCCTACTTCGACG | 164 |
|  | Reverse | CCTGGTTGTCGAGGAAGTCC |  |
| *mexF* | Forward | TGATCCTGATCGTGCCGATG | 145 |
|  | Reverse | GCGAACTCGACGATCAGGAT |  |
| *oprN* | Forward | GGGTCTGTTCAGTCTGCTG | 157 |
|  | Reverse | GGTCGGATCGTCGAACTGTT |  |
| *gyrB* | Forward | CACGTACGACTCTTCCAGCA | 115 |
|  | Reverse | GAACACCATGTGGTGCAGAC |  |
